# Genotype-informed nutrition counselling in clinical practice

**DOI:** 10.1136/bmjnph-2023-000808

**Published:** 2023-12-27

**Authors:** Martin Kohlmeier, Emmanuel Baah, Matthew Washko, Kelly Adams

**Affiliations:** University of North Carolina at Chapel Hill, School of Medicine and Gillings School of Global Public Health, and UNC Nutrition Research Institute, Chapel Hill, NC, USA

**Keywords:** precision nutrition, blood pressure lowering, lipid lowering, irritable bowel syndrome, weight management

## Current knowledge of genotype-specific nutrition responses

Many patients do not achieve the desired response to nutrition interventions because they absorb, metabolise, excrete or regulate food constituents and derived molecules differently than others.^1 2^ The field of research and practice that investigates individual dispositions and seeks to predict likely responses to nutritional interventions is currently called precision nutrition.^3^ In a growing number of evidence-based instances, specific traits are sufficiently understood for use in everyday clinical practice and can be expected to improve the effectiveness of typical dietary treatments. The most common type of trait suggested for individualised nutrition guidance is genetic variation.^4^ Eventually, other types of traits may become actionable, including metabolite signatures, gut microbiome patterns and other biological indicators. Such biological landmarks can assist clinicians in navigating the seemingly endless complexity of nutrition and avoiding the prescription of less effective, one-size-fits-all diets and interventions. It has also become evident that personalised nutrition guidance helps to improve nutrient intakes better than generalised advice.^5^ It is important to emphasise that each algorithmic rule must stand on its own merit and none of them can be used to infer the utility of another one.

## Genotype-informed counselling

A limited list of well-defined and evidence-based nutrigenetic algorithms based on replicated studies is ready now for use in clinical practice to help physicians and other healthcare providers tailor nutrition guidance to the likely needs of some of their patients (table 1). Only algorithms with measurable short-term outcomes have been included. The evidence for each algorithm has been replicated in published studies or corroborated by additional evidence without reporting contradictory findings. The relevant genetic information may be available from commonly used genetic testing companies (23andMe, Ancestry and others). The benefits accrue primarily to the carriers of the specified genotype but much less so or not at all to people without that genotype. Each of the algorithms defines the genetic and other key characteristics of the subgroup the algorithm applies to. The current set of algorithms applies only to adults because sufficient evidence support for children and adolescents is not yet available. The listed algorithms are gender-neutral. The frequencies of the actionable genotypes in Caucasians and African Americans are listed to give a sense of practical relevance. The algorithms apply only to individuals for whom, as a group, there is sufficient supporting evidence. This is currently limited primarily to those of European ancestry and, to some extent, to African Americans due to the limited available supporting evidence.

**Table 1:** Genotype-specific intake guidance

Genotypes	Frequency	Intervention	Counselling instructions (see notes in Appendix 1 for further details)
Obesity: reduce excess body fat			
*APOA2* (CC) (rs5082)	Eu: 14% Afr: 5%	Saturated fat intake under 12 g/day	Reduce your intake of fatty meats, high-fat dairy, chocolates and sweet baked goods. Avoid adding butter or cream to meals or when preparing dishes. ①
*PLIN1* (TT) (rs894160)	Eu: 9% Afr: 10%	Limit carbs	Prefer whole grains, whole-grain breakfast cereals, sweet potatoes, beans and legumes and non-starchy vegetables. ②
*FGF21* (GG)* (rs838147)	Eu: 30% Afr: 49%	Limit fat	Prefer whole grains, whole-grain breakfast cereals, sweet potatoes, beans and legumes and non-starchy vegetables. ②
*FGF21* (AA)* (rs838147)	Eu: 23% Afr: 8%	Limit carbs	Fewer sugary sodas, less bread, pasta, pizza, chips, pretzels, crackers, potatoes, cakes and candy. Eat non-starchy vegetables, nuts and peanuts. ③
High cardiovascular disease risk: reduce hypertension or hyperlipidaemia			
SLC4A5 (AA) (rs7571842)	Eu: 29% Afr: 8%	Sodium <2300 mg	Avoid salted snacks, soups, packaged dressings, pickled foods, bread and canned vegetables. Use spices, herbs and salt substitutes (KCl), not salt. ④
*APOE* (E34 or E44) (rs429358C+rs7412C)	Eu: 20% Afr: 19%	Low saturated fat and cholesterol, low GI	Prefer low-fat foods with a low GI. Whole grains, non-starchy vegetables, nuts and virgin olive or canola oil, similar to Med.Diet. ⑤
*CYP7A1* (GG or GT) (rs3808607)	Eu: 34% Afr: 15%	Soluble fibre beta-glucan	2–3 g beta-glucan is in 1.5 cup cooked oatmeal or in one cup cooked pearl barley or in two teaspoons of psyllium husks. ⑥
*TCF7L2* (TT) (rs7903146)	Eu: 12% Afr: 12%	Med.Diet	Eat primarily plant-based foods, fruits and vegetables, whole grains, legumes, nuts and less meat. Extra virgin olive oil, herbs, spices and less salt. ⑦
Abdominal distress: less indigestible carbohydrates			
*LCT* (CC) (rs4988235)	Eu: 31% Afr: 71%	Limit lactose	Avoid animal milk and soft cheeses; prefer hard cheeses or lactose-free alternatives. Try plant-based products, maybe a lactase supplement. ⑧
*SI* (AA)15Phe (rs9290264)	Eu: 9% Afr: 2%	Limit starch and sucrose	Eat less starches, sugar, maple syrup, sweet fruits and juices (orange, apple, peach, mango, melon, strawberry and pineapple) and milk chocolate. ⑨
Non-alcoholic steatohepatitis: reduce excessive liver fat			
*PNPLA3* (GG) (rs738409)	Eu: 5% Afr: 2%	Limit carbs and lose weight	Limit sugary sodas, bread, pasta, pizza, chips, pretzels, crackers, potatoes, cakes and candy. Use non-starchy vegetables, nuts or peanuts. ③
*MTHFD1* (AA) (rs2236225)	Eu: 20% Afr: 2%	Get enough choline	Good food sources of choline are soy, lecithin, eggs and wheat germ. Consider using a moderately dosed (200–300 mg) dietary supplement. ⑩
Gout: reduce uric acid level			
SLC2A9 (TT) (rs11942223)	Eu: 63% Afr: 39%	Limit fructose	Major sources of fructose are sugar-sweetened beverages, sugars and sweet fruit (strawberry, melon, pineapple, peach, mango, apple and orange). ⑪
Vitamins: improve likely deficient status			
MTHFR (TT) (rs1801133)	Eu: 12% Afr: 6%	Get enough folate	Ensure daily consumption of dark green vegetables, legumes or citrus. Young women should supplement daily with 400 µg of 5-methyltetrahydrofolate (not folic acid). ⑫
GC (TT) (rs4588)	Eu: 5% Afr: NA	Get enough vitamin D	Get 15–30 min of sun per day during the summer while avoiding sunburn. Use a supplement with 2000 IU during other times. More if obese. ⑬
FUT2 (AA) (rs601338)	Eu: 21% Afr: 25%	Get enough vitamin B12	Order a lab assessment of vitamin B12 sufficiency if the patient is over 45. High-dose B12 supplementation with any nitrous oxide exposure. ⑭

*FGF21 rs838147 AA = (rs2307019AA+rs8108136 TT + rs12975781 TT) GG = (rs2307019GG+rs8108136 CC + rs12975781 CC).

Afr, Africa; Eu, Europe; GI, glycaemic index; Med.Diet, Mediterranean diet; NA, not applicable.

### How to use this reference tool

If the genetic information is available, check in the table (left-hand column) whether the patient has a relevant genotype, and then find the intervention (third column from the left) that is probably effective for the condition in this patient. To the right of this column are phrases that can be used to communicate nutrition guidance that is most likely to work for the patient. The words can be embedded in and combined with other relevant advice. We suggest, to be most time effective, using the exact counselling phrases as written on the chart and practising their use with patients before starting to paraphrase and expand on them. Some additional descriptions of these nutrition interventions and practical tips are given in Appendix 1.

## Evidence

### Obesity: reduce excess body fat

Overweight and obesity contribute to many chronic illnesses commonly seen in clinical practice, and reduction of this excess body fat is often desirable to effectively treat conditions such as hyperglycaemia, hyperlipidaemia, hypertension, pain due to arthritis and many others. A familiar problem is that typical weight-loss interventions on their own are ineffective for many patients.^6^ A better path is to match the patient’s genotype with a dietary strategy that is most likely, though not certain, to succeed.^7 8^ Individualised macronutrient patterns should always be used in addition to moderate energy intake reduction and an appropriate exercise programme. For a sizeable minority of patients, it is currently less clear which of numerous alternative macronutrient patterns is most likely to succeed, and for them, we are then still left with generic, one-size-fits-all guidance.

*APOA2*: About one in seven Americans has the rs5082 CC genotype, which is associated with lower weight while consuming minimal amounts of saturated fat but not with higher saturated fat intake.^9^ This interactive effect of saturated fat intake and the rs5082 CC genotype on body weight has been replicated across diverse US, European and Asian populations.^10 11^ Carriers of the CC genotype vulnerable to weight gain tend to have more behavioural traits typically associated with weight gain and higher concentrations of the ‘hunger hormone’ ghrelin with high saturated fat intake than with low saturated fat intake.^11 12^ These effects are not seen in carriers of the TT genotype. Direct interventional confirmation for the genotype-specific effect of saturated fat intake on body weight is currently not available.

*PLIN1*: The common genotype rs894160 TT encodes a form of mediator protein involved in the storage and release of triglycerides into and from adipocytes. The TT genotype is associated with greater weight loss and calorie reduction while consuming a high proportion of carbohydrates (as complex carbohydrates) compared with a low (complex) carbohydrate proportion.^13 14^ Direct interventional evidence is currently not available.

*FGF21*: This gene encodes an important regulator of carbohydrate metabolism and energy utilisation in the liver and other tissues. The rs838147 homozygous genotypes are associated with very distinct dispositions. Carriers of the AA genotype tend to achieve the greatest weight loss during energy restriction when they limit carbohydrates as a percentage of their total (restricted) energy intake, while for those with the GG genotype, a smaller percentage of fat and more (complex) carbohydrates support the weight loss best.^15^ The exact mechanisms underlying this difference require further exploration.

### High cardiovascular disease risk: reduce hypertension or hyperlipidaemia

Multiple mechanisms contribute to an increased risk of myocardial infarction, stroke and related cardiovascular morbidity and mortality. High blood pressure and increased concentrations of atherogenic lipoproteins are not the only risk factors but are known to be important. The following genotype-specific nutrition interventions are just the beginning of targeting them.

*SLC4A5*: Variants in the sodium bicarbonate cotransporter NBCe2, encoded by the *SLC4A5* gene, have been linked to salt sensitivity. Comparison of blood pressure at persistent low versus high salt intake demonstrates that most patients with the rs7571842 AA genotype respond to sodium reduction, while patients with the other genotypes show little or no blood pressure reduction.^16 17^ Systolic blood pressure may be 6–10 mm Hg lower when reducing sodium intake from the typical 3500 mg/day to less than 2300 mg/day, and a medication-sparing effect is often observed.

*APOE*: The haplotype E4 consists of alleles rs429358 C and nearby rs7412 C on the same strand. Carriers of one or two E4 copies can achieve a reduction in elevated low-density lipoprotein (LDL) best by keeping cholesterol and saturated fat intake low while selecting foods that have a low glycaemic index.^18^

Patients with hyperlipidaemia, with the most common combination, APOE E3/3, tend to respond well to standard cholesterol-lowering dietary guidance, including the replacement of saturated fat with monounsaturated fat.^18^ Carriers of the E2 haplotype, particularly those with two E2 copies and dyslipidaemia, often experience better lipid profiles when they can reduce an excess of body fat.^19^ Considering the strong, but individually unreliable,^20^ association of APOE4 with Alzheimer’s disease, the disclosure and discussion of APOE4 carrier status need to be handled very carefully.

*CYP7A1*: LDL cholesterol concentration in blood depends partially on the effective removal of cholesterol from blood circulation and on excretion after CYP7A1-dependent conversion into bile acids. Beta-glucans (a kind of soluble dietary fibre) in some foods (oats, barley and psyllium) bind bile acids in the intestines and promote their excretion with faeces, particularly well in carriers of the common CYP7A1 rs3808607 G allele.^21 22^

*TCF7L2*: Untreated carriers of the rs7903146 TT genotype have a more than twofold increased risk of stroke due to the more frequent presence of hyperglycaemia and other metabolic risk factors.^23^ While most people benefit in various ways from a Mediterranean diet (MedDiet) rich in vegetables and virgin olive oil, this 5-year randomised intervention trial suggested a much lower stroke risk compared with the control (4 vs 10.9 incidence rate per 1000 person-years). Genotype-specific beneficial impacts of the MedDiet on cardiometabolic risk factors were also seen in patients with diabetes and pregnant women.^24 25^

### Abdominal distress: decrease indigestible carbohydrates

*LCT* (lactase): Only a minority of adults (about one-third) around the world express lactase in their small intestines and can digest lactose efficiently. This is because they carry a lactase-persistence variant, such as rs4988235, common in people with European ancestry.^26^ In the absence of such variants, consumption of lactose-containing foods often causes abdominal discomfort and symptoms. The tolerance for such food effects varies greatly between individuals due to differences in expectations, cultural norms and gut microbiome composition. Dietary adjustments are usually effective. Low-lactose food products and supplemental lactase can support the day-to-day management of this predisposition.^27^

*SI* (sucrase/isomaltase): The protein encoded by the *SI* gene and expressed in the brush border of the proximal small intestines is a bifunctional enzyme responsible for digesting sucrose into glucose and fructose and for cleaving the alpha 1>6 bonds of starches that are not broken by amylase or maltase.

Too little sucrase/isomaltase activity in patients with the rs9290264 AA genotype has been shown to be associated with increased stool frequency^28^ and an increased risk of irritable bowel syndrome.^29^

Reducing starch and sucrose intake in patients with sucrase/isomaltase deficiencies decreases symptoms of diarrhoea, constipation and abdominal cramps.^28 30^

### Non-alcoholic steatohepatitis: reduce excessive liver fat

*PNPLA3*: Carriers of the genotype rs738409 GG (148MM) of this gene, which encodes the protein adiponutrin with fat-regulating functions in both adipose and liver, rapidly lose excess liver fat when they limit carbohydrate intake and decrease body fat.^31–34^

*MTHFD1*: The export of fat from the liver depends on the availability of choline to make phospholipids for the construction of the very LDL transport particles. People with the rs2236225 AA genotype have much less capacity for choline synthesis than those with the other genotypes.^35^ Getting enough dietary choline contributes to liver health.^36^

### Gout: reduce hyperuricemia

*SLC2A9*: This gene encodes a transporter that works against renal uric acid excretion. High consumption of fructose increases this urate retention and thereby raises serum uric acid levels in people with the common rs11942223 TT genotype.^37^ In patients with gout who are TT genotype carriers, the resulting higher uric acid levels worsen their condition, while in gout patients with the other genotypes, their gout condition is less affected.^38^ This difference in response opens an opportunity for targeted counselling about avoiding high fructose intake.

### Vitamins: improve likely deficiency

*MTHFR*: Individuals with the genotype rs1801133 TT have severalfold higher folate requirements than people with the much more common CC genotype, based on the changes in plasma homocysteine concentration.^39^ This genotype with a high folate requirement is particularly common in people with Hispanic ancestry. High-dose folic acid supplements lower elevated homocysteine concentrations and reduce the risk of neural tube defects but may be counterproductive in genetically vulnerable individuals due to increased cancer risk.^40^ 5-Methyltetrahydrofolate, the natural form in dark-green vegetables, citrus and legumes, can be used instead.^41 42^

*GC*: The common genotype rs4588 TT is consistently associated both in healthy children and adults with lower-than-average vitamin D concentrations in blood in the USA, Europe and other regions.^43^ Cohort studies and clinical trials found multiple health benefits of vitamin D supplementation in rs4588 TT genotype carriers. Such benefits included fewer colorectal adenomas,^44^ better blood pressure control^45^ and more.

*FUT2*: The genotype rs601338 AA identifies individuals who are Lewis (ABO) blood type non-secretors and at increased risk of vitamin B12 deficiency. Vitamin B12 supplementation has been shown to be effective. This is regardless of whether increased vulnerability of the gastric epithelial lining to *Helicobacter pylori* infection with loss of intrinsic factor production^46^ is primarily responsible or a difference in hepatobiliary vitamin B12 recovery.^47^

## Summary and perspective

Several genotype-specific algorithms for nutrition guidance are supported by robust evidence and can be applied in everyday medical practice to improve the outcomes of patients with common clinical conditions. Because an increasing number of patients already have personal genotype information from popular applications, it is often worthwhile to ask patients whether they want to share their genotype data and explain some practical uses and consequences. It is important to recognise that the interactions are probabilistic in nature and will not apply to every single patient, just to most of them.

The efficacy of some familiar nutrition interventions can be greatly boosted when directed only to patients who are likely to benefit and not suggested for likely non-responders. A targeted approach to nutrition guidance will save time and frustration for both patients and providers.

10.1136/bmjnph-2023-000808.supp1 Supplementary data
