# Supplementary material for: Genotype-informed nutrition counselling in clinical practice
Source: BMJ Nutr Prev Health. 2023 Dec 27;6(2):407–12. doi: 10.1136/bmjnph-2023-000808 (PMC11009529; doi:10.1136/bmjnph-2023-000808)
Supplement: Supplementary data [file bmjnph-2023-000808supp001.pdf]

Appendix 1: Notes for genotype-specific dietary counseling

① *Minimal saturated fat intake*

In addition to calorie reduction and an exercise program, reduce intake of fatty meats, high-fat dairy, chocolates, sweet baked goods. Avoid adding butter/cream to meals or for preparing dishes.

Plant-based intake patterns can help to limit saturated fat consumption if attention is paid to fat sources. Unfavorable plant fats include palm oil, palm kernel oil, and coconut oil.

② *Emphasis on complex carbohydrate intake*

In addition to calorie reduction and an exercise program, prefer whole grains, whole-grain breakfast cereals, sweet potatoes, beans & legumes, and non-starchy vegetables.

All carbohydrates combined should provide 55 or more percent of total, possibly reduced, energy intake. Simple sugars should be used sparingly, as occasional treats.

③ *Limit carbohydrate intake*

In addition to calorie reduction and an exercise program, limit intake of sugary sodas, bread, pasta, pizza, chips, pretzels, crackers, potatoes, cakes, candy. Eat non-starchy vegetables, nuts/peanuts.

Patients can follow a low-carbohydrate meal plan by limiting grains and starchy vegetables (e.g., corn, lima beans, peas, potatoes, winter squash), while leaning more heavily on non-starchy vegetables, such as leafy greens, peppers, carrots, summer squash, and cruciferous vegetables (broccoli, cauliflower, Brussels sprouts, cabbage). Reading food labels is important since many foods contain added sugars and starches. Frequent use of non-caloric sweeteners has its own challenges and should not be the only solution. Sugar sensors in the gut respond to some extent also to sugar substitutes. Non-nutritive sweeteners also have specific and potentially undesirable effects on the gut microbiome [1].

④ *Reduced sodium consumption*

Keep sodium intake well under 2300 mg/day. Avoid salted snacks, soups, packaged dressings, pickled foods, most canned vegetables (frozen vegetables are desirable and as good as fresh ones). Consume bread sparingly because it usually contains a lot of added salt. Use spices and herbs generously. Salt substitutes (such as potassium chloride, KCl) can be considered instead of salt, but many find the slightly bitter flavor unpleasant. The potential risk of hyperkalemia for some patients may play a role.

Sodium intake can be limited without too much effort by avoiding salted foods like pretzels, nuts, chips, French fries, and soups. Significant amounts of sodium (read food labels) are also in many pasta sauces, ramen noodles, pizza, most breads and salad dressings, pickled foods, processed meats, and prepackaged, frozen, or restaurant meals. Canned beans and vegetables also tend to be high in sodium. Switching from a meat-based diet to a more plant-based food pattern is often helpful. Spices and herbs give dishes flavor and should replace table salt and monosodium glutamate (MSG) and similar industrial flavor enhancers.

⑤ *Low GI diet and low intake of saturated fat and cholesterol*

Prefer low-fat foods with low glycemic index (low GI). Use whole grains, non-starchy vegetables, nuts, virgin olive/canola oil. The pattern is like that of a Mediterranean Diet.

Legumes, brown rice, and whole wheat pasta cooked ‘al dente’ are complex carbohydrates that have a low GI. Patients can be encouraged to explore foods and dishes with diverse culinary options that appeal to them.

⑥ *High beta glucan intake*

2-3 g beta-glucan is in 1.5 cup cooked oatmeal, or in 1 cup cooked pearl barley, or in 2 teaspoons of psyllium husks. Rye, sorghum, baker’s yeast, seaweed, brown algae, and some mushrooms, including oyster, lion’s mane, maitake, shiitake, and reishi mushrooms, are also good sources.

Beta-glucan-rich foods can be incorporated into appealing burgers, stews, soups, smoothies, and baked goods.

⑦ *Mediterranean Diet*

Eat primarily plant-based foods, fruits/vegetables, whole grains, legumes, nuts, less meat. Extra virgin olive oil, herbs, spices, less salt.

The Mediterranean Diet is a predominantly plant-based eating pattern that contains abundant fruits, vegetables, legumes, and whole grains (e.g., brown rice, bulgur, oats, quinoa, whole wheat). Seafood is the preferred form of animal protein. Red meat and dairy are used sparingly. Olive oil is the preferred form of added fat, instead of butter, margarine, or other oils. There are various patterns that conform with the broader concept [2]. The extent to which these modifications replicate the benefits of the original pattern rich in fruit, vegetable, and olive oil bioactives, is less certain.

⑧ *Low-lactose and lactose-free choices*

Avoid animal milk and soft cheeses, prefer hard cheeses or lactose-free alternatives. Try plant-based products, maybe a lactase supplement.

An increasing number of dairy products and other foods are now available that have been treated with lactase and therefore lactose-free. A similar effect can be achieved by some patients when they ingest a lactase supplement (tablet or liquid) before enjoying their favorite lactose-containing food. Many fermented dairy products have naturally reduced lactose content due to the microbial action. Yogurts, kefirs, and well-aged cheeses often work for patients with lactose intolerance.

⑨ *Limit starch and sucrose consumption*

Limit starches, sugar, maple syrup, and sweet fruits & juices (orange, apple, peach, mango, melon, strawberry, pineapple), milk chocolate, dulce de leche.

Starches are plentiful in pasta, potato, bread, and pastries. The consumption of these foods should be moderated in favor of non-starchy vegetables and legumes. Sucrose (mostly from sugar cane and sugar beets) is added to many foods generally perceived as healthy, such as yogurt, flavored milk, and canned fruit. Limiting specifically sucrose requires careful attention to food labeling and ingredient lists. Discussion of individual sensitivity to starches and sucrose can offer an opportunity to promote a healthier eating pattern with less sugars and low-glycemic-index foods overall.

⑩ *Adequate choline intake*

Good food sources of choline are soy, lecithin, eggs, wheat germ. Consider using additionally a moderately dosed (200-300 mg) dietary supplement.

Whether excessive choline intake, such as with multiple eggs a day or with high-dosed supplements carries its own risk due to conversion of choline by gut microbes to the putative risk factor TMA/TMAO remains to be seen [3].

⑪ *Limit fructose intake*

Major sources of fructose are sugar-sweetened beverages, sugars, sweet fruit (strawberry, melon, pineapple, peach, mango, apple, orange).

Education and guidance of vulnerable individuals can focus on the fact that not only sweetened beverages and candy, but also excessive consumption of sweet fruit can trigger gout episodes.

⑫ *Get enough folate*

Ensure daily consumption of dark green vegs, legumes, or citrus. Young women should supplement daily with 400 µg MTHF (not folic acid).

Some people, those with low-activity DHFR activity, are at risk of accumulating folic acid when getting it with a high-dosed supplement, linked to increased breast cancer risk and other adverse health outcomes.

⑬ *Get enough vitamin D*

Get 15-30 minutes sun per day during sunny season while avoiding (even minor) sunburn. Use a dietary supplement with 2000 IU during other times. More will be needed if the patient is obese.

Increased vitamin D deficiency risk is associated with living at high latitude (north of Washington, DC, about 39 degrees North), regular use of sun protection, and confinement to indoor living.

⑭ *Get enough vitamin B12*

Order a lab assessment of vitamin B12 sufficiency if the patient is over 45 years old. Directly supply a B12 supplement to patients who had nitrous oxide exposure (from medical procedures or for recreational purposes) with a high dose (typically 1000 µg vitamin B12 as a single dose).

There are no known risks from high vitamin B12 intake, but also no additional benefits.

REFERENCES

1. Conz A, Salmona M, Diomede L. Effect of non-nutritive sweeteners on the gut Microbiota. *Nutrients* 2023;15:1869.

2. Handu D, Piemonte T. Dietary approaches and health outcomes: an evidence analysis center scoping review. *J Acad Nutr Diet* 2022;122:1375–93.

3. Meyer KA. Population studies of TMAO and its precursors may help elucidate mechanisms. *Am J Clin Nutr* 2020;111:1115–6.
